# Giving people the words to say no leads them to feel freer to say yes

**DOI:** 10.1038/s41598-023-50532-3

**Published:** 2024-01-05

**Authors:** Rachel Schlund, Roseanna Sommers, Vanessa K. Bohns

**Affiliations:** 1 Department of Organizational Behavior, School of Industrial and Labor Relations, Cornell University, Ithaca, NY USA; 2 University of Michigan Law School, Ann Arbor, MI USA

**Keywords:** Psychology, Human behaviour

## Abstract

We examine how to structure requests to help people feel they can say no (or yes) more voluntarily. Specifically, we examine the effect of having the requester provide the request-target with an explicit phrase they can use to decline requests. Part of the difficulty of saying no is finding the words to do so when put on the spot. Providing individuals with an explicit script they can use to decline a request may help override implicit scripts and norms of politeness that generally dictate compliance. This should make individuals feel more comfortable refusing requests and make agreement feel more voluntary. Hence, we hypothesized that telling people *how* to say no (by providing them with an explicit script) would make compliance decisions feel more voluntary above and beyond merely telling them they *can* say no. Across two experimental lab studies (*N* = 535), we find support for this prediction.

## Introduction

Not all compliance is equal. Although the behavior appears the same from the outside, people can agree more or less voluntarily with a request. In other words, they can enthusiastically consent or reluctantly comply^1,2^, give or give in^3,4^.

It is advantageous to both requesters and request targets to ensure that compliance is not simply the result of giving in to social pressure. Targets who feel forced or “guilted” into complying with requests can feel regret, frustration, helplessness^5^, and resentment^6–8^. Ultimately, they may provide lower-quality help^4,9^ or back out of their commitments at a less convenient time for the requester once their obligations become more concrete.^10^ Moreover, both requesters and targets may find themselves in uncomfortable situations if targets begrudgingly agree to problematic romantic^11,12^ or unethical^13^ requests.

All of this suggests numerous benefits for both parties in identifying methods of soliciting voluntary consent, not mere acquiescence or compliance^1,2^. Yet, this is difficult to accomplish. People often agree with requests—even those they would rather refuse—because it is so hard to say no. Saying no is a face-threatening act^14,15^, which can feel deeply uncomfortable and socially risky^16,17^. Denying a request risks insinuating something negative about the requester or their request^18^. Ultimately, targets of requests feel obligated to follow implicit scripts and norms of politeness and respect that presume compliance^19–22^.

Considerable research has explored strategies to help targets overcome the difficulties of saying no, such as the use of self-affirmations^23^, refusal frames^24,25^, and “positive no’s,” or “yes, no, yes” strategies^26^, among others^5,27,28^. However, such interventions place the burden of saying no, while maintaining face, entirely on targets. Further, these interventions can be questionably effective and impractical, often requiring targets to come up with elaborate multi-part responses^26^ that are unrealistic to implement when targets are put on the spot in the moment.

As an alternative approach, the current research examines strategies requesters can use to formulate requests that, rather than merely secure compliance, leave targets feeling able to make their own genuinely voluntary choices. One common strategy requesters use is to add something along the lines of “but you are free to say no” at the end of a request. However, research finds that “verbally recognizing the target’s freedom to say ‘no’” tends to *increase* compliance^29^ and that emphasizing the target’s “right to refuse” has little effect on targets’ feelings of freedom to say no^30^.

We hypothesize that statements that seek to reassure targets about the lack of material consequences of refusal (e.g., no penalty for refusal) fail to address targets’ concerns about the possible *social* consequences of refusal—namely, the possibility that they will offend their interaction partner by violating norms of politeness and respect^19–21^. Thus, statements emphasizing that “you are free to accept or refuse” or that you have “the right to refuse” fail to dispel the implicit scripts that demand cooperation in the form of compliance. In other words, even if targets are reassured that they *can* say no, the real problem is knowing *how* to do so graciously.

Although research suggests that using an indirect communication channel, such as email, can elicit more voluntary compliance^31–33^, we are focused on face-to-face requests, precisely because they are more difficult to decline. Finding the words to graciously refuse a request (and words that targets know requesters perceive as gracious) may be especially difficult when one is put on the spot during a face-to-face request.

Therefore, the current research tests an intervention in which requesters provide targets with information about *how* to say no, thereby supplanting, or at least diminishing the power of, the implicit script dictating compliance with an explicit script offering a (still polite) alternative. Specifically, participants in two lab experiments were asked to respond to a highly intrusive request—one most people say they would prefer to decline^30^—using one of two scripts. In one, requesters assured targets that they could refuse the request. In the other, they provided targets with specific words to communicate refusal. In both studies, the latter intervention led participants to experience their decision as more voluntary—that is, participants reported feeling freer to say no.

## Open practices and data availability statement

In all studies, the sample size, or data collection stopping point, was determined before data collection began, and all analyses were two-tailed tests performed after data collection was completed. We report all variables, manipulations, measures, data exclusions, and sample size rationales. All studies (except for the pilot study) were pre-registered, and the data, code, materials, and pre-registrations for each study are available on the Open Science Framework (OSF; https://osf.io/vs3rh/?view_only=1b3baadc6f4e4fe4a30a8089f11b83b7). We report all pre-registered results. Here we reverse the order in which our pre-registration discusses our two key dependent measures (behavioral compliance and freedom to say no). This is due to the fact that our results yield stronger conclusions about how the intervention affected feelings of freedom than about how it influenced behavioral compliance. However, there were no deviations from our pre-registered analyses or predictions. This research was conducted in accordance with established ethical guidelines and was approved by the Institutional Review Board at Cornell University.

## Study 1: Can an intervention increase how free targets feel responding to an intrusive request?

To investigate whether an intervention on the request side could increase how free people feel to say no, we asked participants to unlock their password-protected smartphones and hand them over to a student experimenter to look through in another room. Although this kind of request may sound outrageous, in reality, people regularly agree to such requests. For instance, people agree to hand over their passwords and other sensitive data to social engineers^34,35^ and even engage in acts of vandalism at others’ behest^13^.

In previous studies using this paradigm^30,36^, participants overwhelmingly complied with this request while simultaneously reporting they would prefer to refrain from complying. Importantly, explicitly advising participants they could refuse the search without penalty did not increase their feelings of freedom to decline the request in these studies. In the current study, we attempted to increase participants’ subjective feelings of freedom by offering them specific language they could use to communicate refusal.

## Method

### Participants

We recruited 198 adult participants from a large research university in the United States. The participants completed the study as an opportunity for extra credit in participating courses. As it was not possible to determine how many participants would choose to participate, we aimed to collect as many participants as possible within a pre-registered, pre-determined period (i.e., until the end of the fall semester). Based on our pre-registered exclusion criteria, we excluded participants who (a) did not have a phone, (b) had an inaccessible phone (e.g., out of batteries, set to a language other than English), (c) recognized the experimenter from another context, or (d) reported having heard details of the study before participating, yielding a final sample of 174 participants (58% identified as women, 40.2% as men, and 1.7% as non-binary/gender non-conforming; 5.2% as Black/African American, 32.2% as Asian/Asian American/Pacific Islander, 42% as White/European American, 4% as Latino/Hispanic American, 0.6% as Middle Eastern/Arab American, and 16% as Biracial/Mixed-Race; *M*_age_ = 18.75, *SD*_age_ = 1.98). A sensitivity analysis using the *pwr* R package^37^ revealed that this sample size carries an 80% chance of detecting effect sizes of *d* = 0.43 and Φ = 0.21 (or larger) for each dependent variable (behavioral compliance and freedom to say no).

### Procedure

We randomly assigned participants to one of two conditions: the “right to refuse” consent request (control) condition, which informed participants of their right to refuse, or the “how to refuse” consent request (intervention) condition, which offered participants a script informing them *how* to refuse. Specifically, all participants arrived at the lab and were greeted by an experimenter, who was always one of two undergraduate research assistants unaware of the hypothesis and trained to follow a script. To participants in the “right to refuse” condition (*n* = 86), the experimenter stated (differences between conditions are indicated in italics),

Before we begin the study, can you please unlock your phone and hand it to me? I’ll just need to take your phone outside of the room for a moment to check for some things. *If you’d like to refuse, you may do so.* Refusing will not affect your payment or participation in the study.

To participants in the “how to refuse” condition (*n* = 88), the experimenter stated,

Before we begin the study, can you please unlock your phone and hand it to me? I’ll just need to take your phone outside of the room for a moment to check for some things. *If you’d like to refuse, please say the words, ‘I’d rather not.’* Refusing will not affect your payment or participation in the study.

After delivering the script, the experimenter held out a small basket and waited for the research participant to hand over their phone. If the participant surrendered their unlocked phone, the experimenter took it out of the room and waited five seconds before re-entering the room and returning it. If the participant declined to allow the search, the experimenter immediately moved on to the next phase of the study.

Next, participants filled out a questionnaire that included a series of questions that together comprised our measure of how free they felt to say “no” to the request. Specifically, participants were asked, “How easy was it/would it have been to say ‘no’ to this request?” “How comfortable did you feel/would you have felt saying ‘no’ to this request?” and “How free did you feel/would you have felt saying ‘no’ to this request?” (α = 0.80) and responded on a 7-point Likert scale ranging from 1 (*Not at all*) to 7 (*Extremely*). While the participants filled out the questionnaire (see OSF for full materials), the experimenter filled out a form indicating whether the participant had complied with the request, defined as handing over their unlocked phone. Importantly, and following our Institutional Review Board’s approval, participants consented to have their data used *after* they responded to the request to hand over their phone to avoid any confounds that may emerge from obtaining informed consent beforehand.

## Results

**Figure 1:**
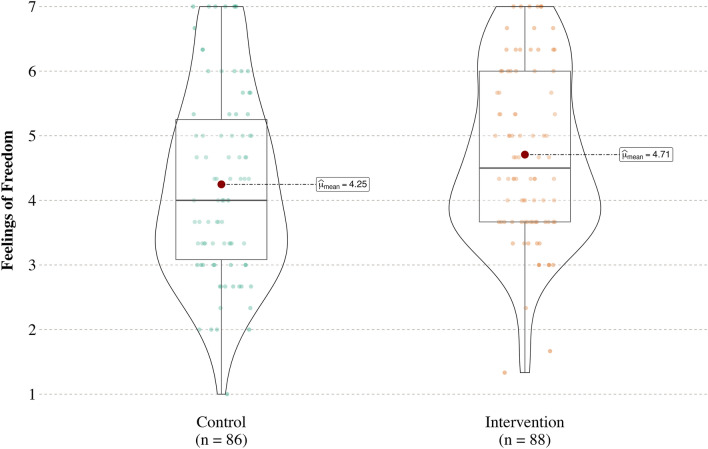
Figure 1 presents a violin plot of feelings of freedom by condition in Study 1. The center dot identified by a text box reflects the mean, and the box surrounding it is a box plot with vertical lines above and below the box plot representing the interquartile range. The shape of each violin corresponds to the distribution of the data.

As predicted, participants felt freer to say no in the “how to refuse” (intervention) condition (*M* = 4.71, *SD* = 1.31), 95% CI [4.43, 4.99], than in the “right to refuse” (control) condition (*M* = 4.25, *SD* = 1.45), 95% CI [3.94, 4.56], *t*(172) = 2.20, *p* = 0.029, *d* = 0.33, 95% CI [0.03, 0.63] (see Fig. 1).

We did not find a significant difference in the number of participants who complied with the request in the “how to refuse” condition (70%) as compared to the “right to refuse” condition (80%), *χ2*(1, *N* = 174) = 2.24, *p* = 0.135, Φ = 0.11, 95% CI [–0.04, 0.26].

## Study 2: Higher powered replication

While the intervention in Study 1 effectively increased participants’ feelings of freedom to say no, the intervention’s effect on behavioral compliance was less clear. The difference between conditions was not significant; while fewer participants complied with the request in the intervention condition, the difference was not large enough to rule out a null effect. At the same time, the study lacked the power to conclude that the intervention had *no* effect on behavior. Thus, in Study 2, we recruited a larger sample of participants and modified the script to increase the contrast between the two conditions. We conducted a pilot study to determine the appropriate sample size for Study 2 using this modified script (reported below).

### Pilot study

We recruited 40 participants from a large research university in the United States. Because this was a pilot study, we aimed to recruit 25 participants per cell. We followed our four pre-registered exclusion criteria from Study 1, yielding a final sample of 38 participants (73.7% identified as women, 21.1% as men, and 5.2% did not report on their gender; 7.9% as Black/African American, 55.3% as Asian/Asian American/Pacific Islander, 31.6% as White/European American, and 5.2% as Biracial/Mixed-Race; *M*_age_ = 23.24, *SD*_age_ = 5.14). A sensitivity analysis using the *pwr* R package^37^ revealed that this sample size carries an 80% chance of detecting effect sizes of *d* = 0.94 and Φ = 0.46 (or larger) for each dependent variable.

The procedure of the pilot mirrored that of Study 1, except we revised the experimenter’s request to differentiate the conditions more starkly. The new scripts were designed to reinforce the manipulation by referring to the key sentence earlier in the script and in the last sentence while keeping the manipulation subtle enough to avoid demand effects. Importantly, as in Study 1, participants gave informed consent *after* responding to the main request. The modified “right to refuse” (control) condition script stated:

Before we begin the study, can you please unlock your phone and hand it to me? I’ll just need to take your phone outside of the room for a moment to check for some things. As a participant in a research study, you have the right to refuse at any time. *I will tell you more about your right to refuse if you choose to.* Refusing will not affect your payment or participation in the study. *If you would like to refuse, you may do so now*.

The modified “how to refuse” (intervention) condition script stated:

Before we begin the study, can you please unlock your phone and hand it to me? I’ll just need to take your phone outside of the room for a moment to check for some things. As a participant in a research study, you have the right to refuse at any time. *I will tell you more about how you can refuse if you choose to.* Refusing will not affect your payment or participation in the study. *If you would like to refuse, you can let me know now by saying the words, “I’d rather not,” or “No, thank you.”*

As in Study 1, after reading the appropriate script for the given condition, the experimenter held out a small basket and waited for the participant to hand over their phone.

We randomly assigned a total of 19 participants to the “right to refuse” (control) condition and 19 participants to the “how to refuse” (intervention) condition. Replicating the results of Study 1, participants felt freer to say no in the “how to refuse” condition (*M* = 4.89, *SD* = 1.15), 95% CI [4.34, 5.45], then in the “right to refuse” condition (*M* = 3.88, *SD* = 1.45), 95% CI [3.18, 4.57], *t*(36) = 2.40, *p* = 0.022, *d* = 0.78, 95% CI [0.10, 1.46]. Further, in this pilot study, fewer participants complied with the request in the “how to refuse” condition (58%) than in the “right to refuse” condition (95%), *χ2*(1, *N* = 38) = 7.13, *p* = 0.008, Φ = 0.43, 95% CI [0.12, 0.75].These pilot results provided evidence that the revised scripts effectively increased the strength of the manipulation. We used these results in combination with the results from Study 1 to determine the sample size in Study 2.

## Method

### Participants

We recruited 345 participants from two large research universities in the United States. Based on the effect sizes observed in Study 1 and the pilot study (along with resource constraints), we aimed to recruit 300 participants (150 participants per cell). Specifically, we pre-registered that we would continue to recruit participants until we reached our target sample size or until May 19, 2023 (the end of the spring semester), whichever came first. Applying our pre-registered exclusion criteria resulted in a final sample of 323 participants (68.1% identified as women, 28.7% as men, 0.3% as transgender, 1.9% as non-binary/gender non-conforming, and 1% preferred not to respond regarding their gender; 5.3% as Black/African American, 45.5% as Asian/Asian American/Pacific Islander, 32.8% as White/European American, 4.3% as Latino/Hispanic American, 1.2% as Middle Eastern/Arab American, and 10.9% as Biracial/Mixed-Race;* M*_age_ = 22.63, *SD*_age_ = 6.43). A sensitivity analysis using the *pwr* R package^37^ revealed that this sample size has an 80% chance of detecting effect sizes of *d* = 0.32 and Φ = 0.16 (or larger) for each dependent variable. The procedure in Study 2 was identical to that in the pilot study. Consistent with our pre-registration, we do not include pilot study participants in the results reported here.

## Results

**Figure 2:**
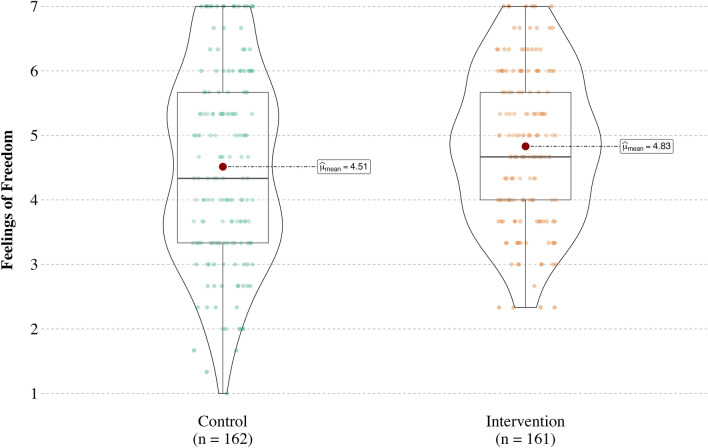
Figure 2 presents a violin plot of feelings of freedom by condition in Study 2. The center dot identified by a text box reflects the mean, and the box surrounding it is a box plot with vertical lines above and below the box plot representing the interquartile range. The shape of each violin corresponds to the distribution of the data.

As predicted and replicating the results from Study 1, participants felt freer to say no in the “how to refuse” (intervention) condition (*M* = 4.83, *SD* = 1.17), 95% CI [4.65, 5.01], than in the “right to refuse” (control) condition (*M* = 4.51, *SD* = 1.47), 95% CI [4.29, 4.74], *t*(321) = 2.14, *p* = 0.033, *d* = 0.24, 95% CI [0.02, 0.46] (see Fig. 2).

Once again, the difference in behavioral compliance between the “how to refuse” condition (78%) and the “right to refuse” condition (83%) was not statistically significant, *χ2*(1, *N* = 323) = 1.67, *p* = 0.197, Φ = 0.072, 95% CI = [–0.04, 0.18]. However, as the effect was ultimately smaller than we had estimated from our pilot study, we were once again underpowered to conclusively determine that there was *no* effect on behavior compliance. 

To investigate why the pilot results differed from the Study 2 results, which used the same materials and procedure, we ran several analyses on the demographic variables we collected (the only individual difference variables that were included in the study). These analyses did not reveal any differences in demographic composition between the conditions within each study and were ultimately unable to explain the difference in effect size. These exploratory analyses are reported in the SOM.

To try to identify the size of the effect more precisely^38,39^, we conducted an internal meta-analysis across the studies for each dependent variable. In the main text, we report the results of these internal meta-analyses which include Studies 1 and 2 and omit the pilot study (out of concern that the pilot study may be subject to small sample size bias). In the SOM, we report these same analyses with all available data, including the pilot study. When the pilot study data is included, the test for the overall effect is significant for both dependent variables (see SOM).

## Internal meta-analysis

Study 2 replicated the finding from Study 1—telling participants *how* to refuse led participants to feel freer to refuse than telling participants they had the *right* to refuse. As described earlier, this represents significant progress in the effort to design choice environments that leave people feeling genuinely able to make a free choice. If compliance is to be a meaningful expression of people’s felt preferences rather than the product of a decision environment that—while providing nominal choice—ultimately limits how free people really feel to act on their wishes, identifying interventions that improve feelings of freedom is paramount.

Still, Study 2 left us unable to conclusively settle the question of whether the “how to refuse” intervention affects compliance *behavior* in addition to subjective feelings of freedom. Although we based Study 2’s sample size on Study 1 and a pilot study in which compliance behavior was significantly affected by the intervention, our pre-registered study again yielded an effect that was too small to rule out the null hypothesis that the intervention did not affect behavior.

Thus, it appears that if this intervention alters compliance behavior, the effect is likely to be small. Notably, an intervention that increases subjective voluntariness (as indexed by the feelings of freedom dependent measure) while simultaneously leaving rates of compliance unaffected (as measured by the behavioral compliance measure) is likely to be viewed favorably by practitioners, such as a charitable organization that seeks to solicit donations in a low-pressure manner (which leaves potential donors feeling genuinely free to refuse) without significantly decreasing donation rates.

In an attempt to draw a more conclusive answer to the question of whether the intervention affects compliance behavior, we conducted an internal meta-analysis of Studies 1 and 2 on both dependent measures (freedom to say no and behavioral compliance). Internal meta-analyses can be helpful when the effect size detected is small and individual studies may be underpowered^38^, and they can also increase the precision of estimates^39^.

**Figure 3:**
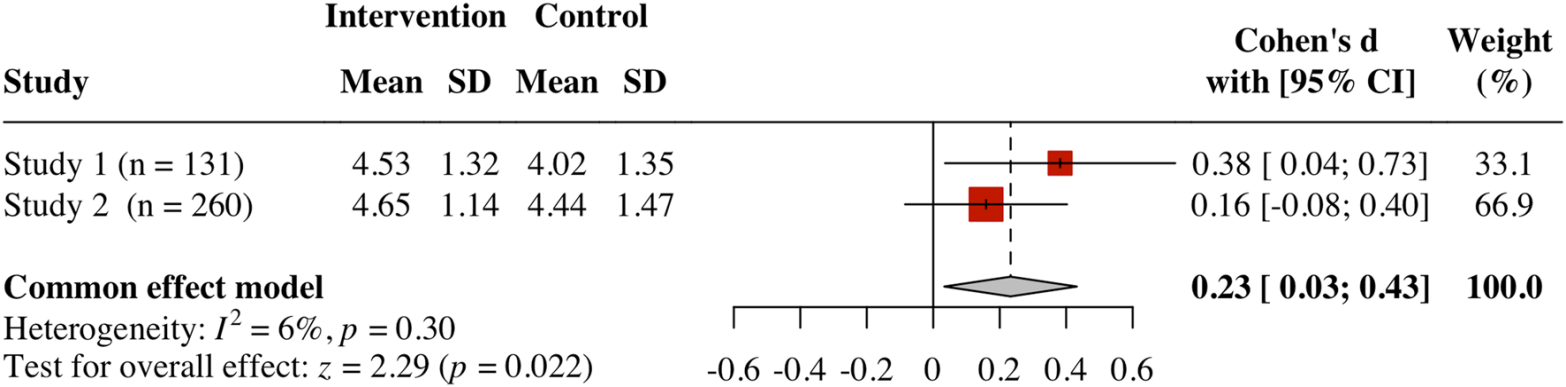
Forest plot of feelings of freedom across studies.

Using the *metafor* R package^40^, we first meta-analyzed the results of our primary measure of interest: participants’ feelings of freedom to refuse the request. Across all studies, participants in the “how to refuse” (intervention) condition felt freer to say no than participants in the “right to refuse” (control) condition, *d* = 0.27, *Z* = 3.03, *p* = 0.002, 95% CI [0.10, 0.45] (see Fig. 3).

**Figure 4:**
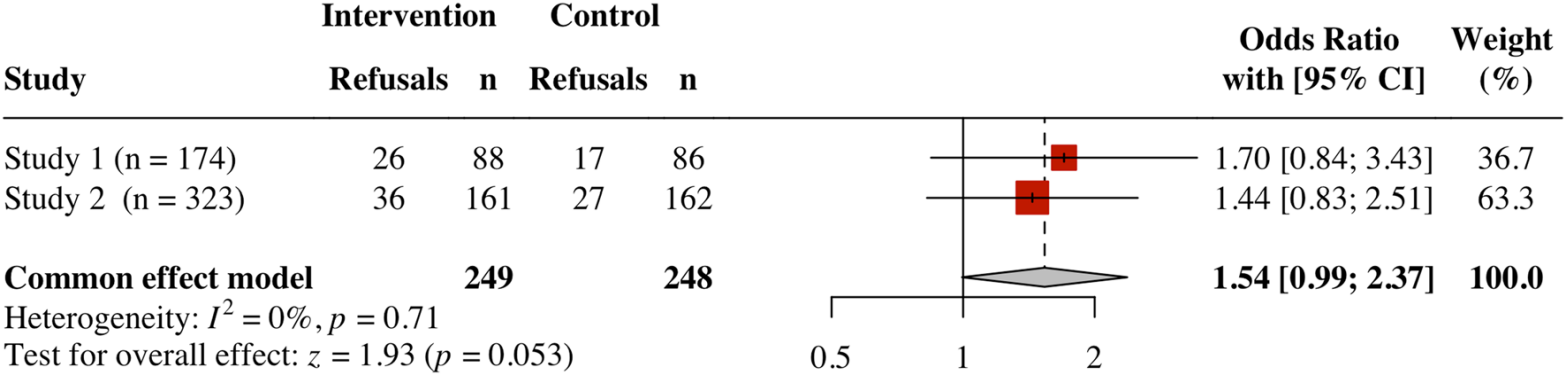
Forest plot of behavioral compliance across studies.

We then meta-analyzed the results of the behavioral compliance measure. Collapsed across Studies 1 and 2 (but not including the pilot study participants), the effect by condition on behavioral compliance was non-significant, *OR* = 1.54, *Z* = 1.93, *p* = 0.053, 95% CI [0.99, 2.37] (see Fig. 4).

In sum, in these meta-analyses, the effect on behavioral compliance is not significant (*p* = 0.053), but the effect on subjective voluntariness (feelings of freedom to say no) appears robust.

## General discussion

We identified a way of structuring requests that allows requesters to ask for what they want while simultaneously decreasing the social pressure on targets to comply. Specifically, in two pre-registered, experimental lab studies, we found that providing request targets with information about *how* to communicate refusal rather than simply reassuring them that they *can* refuse made targets feel freer to decide whether to agree with a request. Unlike simply reassuring targets of their right to refuse, offering targets specific language they can use to communicate refusal likely overrides implicit scripts that dictate compliance^19–21^. Consequently, targets feel more comfortable refusing requests, making their decision of whether or not to comply feel more voluntary.

Unlike previous research on social influence and compliance^19,41,42^, our findings do not necessarily carry clear implications for *behavioral* compliance, but rather suggest implications for the *psychology underlying* compliance, or the extent to which a target feels as if saying no is a genuinely available option. This is important because the same behavior—compliance with a request—can be experienced as more or less voluntary, which carries implications for distinguishing genuine consent from mere compliance^1,2^.

The current research also extends past work examining strategies to help request targets overcome the difficulties of saying no^5,27,28^. Research in this area has focused on tactics targets must deploy to overcome the difficulties of saying no. In contrast, our intervention represents a tactic requesters can deploy in seeking to ensure voluntary agreement rather than acquiescence.

There are several avenues for future research that build on the current findings. For one, these studies did not manipulate relational or power dynamics between requesters and targets, which may be fruitful avenues for future research. Both power^43^ and status^44^ have been found to increase compliance with requests, suggesting they could be potential moderators of our effect. For example, it is possible that when request targets hold less power than requesters, overriding politeness norms with a script of how to say no may prove inadequate to overcome the pressure lower-power individuals feel to acquiesce. On the flip side, research has shown that power disinhibits the pressure people feel to abide by social norms and scripts^45,46^. These disinhibiting effects may make such interventions unnecessary for high-power request targets, who may already feel empowered to say no.

In addition, people’s experience of compliance as more or less voluntary may have important downstream consequences. For example, as theorized by Bohns & Schlund (2020), feeling pressured to comply with workplace requests may erode a sense of fairness, autonomy, and trust in the organization. More broadly, feeling as if one has no choice but to comply may lead to feelings of regret. Future research should investigate these and other possible downstream consequences that may result from securing (or failing to secure) voluntary compliance.

### Supplementary Information

Supplementary Information.
