# Supplementary material for: Giving people the words to say no leads them to feel freer to say yes
Source: Sci Rep. 2024 Jan 5;14:576. doi: 10.1038/s41598-023-50532-3 (PMC10770178; doi:10.1038/s41598-023-50532-3)
Supplement: Supplementary file 1 — Supplementary Information. [file 41598_2023_50532_MOESM1_ESM.docx]

**Supplemental Online Materials For:**

**Giving people the words to say no leads them to feel freer to say yes**

Rachel Schlund ^a^, Roseanna Sommers ^b^, & Vanessa K. Bohns ^a^

^a^ Cornell University, Department of Organizational Behavior

^b^ University of Michigan, School of Law

***Corresponding author:** Rachel Schlund

**This document includes:**

**Table of Contents**

[**Study 1: Can an Intervention Increase How Free Targets Feel 2**](#_Toc138754123)

[**in Response to an Intrusive Request? 2**](#_Toc138754124)

[**Study 1: Additional Analyses** 2](#_Toc138754125)

[**Study 2: Higher Powered Replication 2**](#_Toc138754126)

[**Study 2 (Pilot): Additional Analyses** 2](#_Toc138754127)

[**Study 2: Additional Analyses** 3](#_Toc138754128)

[**Internal Meta-Analysis: Including Pilot Data 4**](#_Toc138754129)

**Study 1: Can an Intervention Increase How Free Targets Feel**

**in Response to an Intrusive Request?**

**Study 1: Additional Analyses**

One of the scale items in Study 1 contained an error, which changed the meaning of the scale item. Specifically, the item asked participants, “How pressured did you feel/would you have felt to say “no” to the request?” The item was supposed to ask, “How pressured did you feel/would you have felt to say “yes” to the request?” However, removing or including the item does not substantially change the results. Thus, in the main text, we report the results without the item included in the scale composite, and here, in the SOM, we report the results with the item included in the scale composite.

When including the scale item in the scale composite, as predicted, participants felt freer to say no in the “how to refuse” (intervention) condition, *M* = 4.76, *SD* = 1.26, 95% confidence interval (CI) = [4.49, 5.02], then in the “right to refuse” (control) condition, *M* = 4.35, *SD* = 1.26, 95% CI = [4.08, 4.62], *t*(172) = 2.12, *p* =.035, *d* = .32, 95% CI = [0.02, 0.62].

Further, we observe no difference by condition in participants’ self-identified gender, *χ2*(1, *N* = 2) = 0.62, *p* = .735, Φ = 0.06, 95% CI = [-0.09, 0.21], or ethnicity, *χ2*(1, *N* = 5) = 2.00, *p* = .850, Φ = 0.11, 95% CI = [-0.04, 0.26]. Additionally, we conducted a likelihood ratio test investigating the effect of gender in addition to the effect of condition on compliance, which revealed that it was not a significantly better predictor, *χ2*(1, *N* = 2) = 3.41, *p* = .182. The results of a separate likelihood ratio test investigating the effect of ethnicity in addition to the effect of condition on compliance also revealed that it was not a significantly better predictor, *χ2*(1, *N* = 5) = 2.07, *p* = .839.

**Study 2: Higher Powered Replication**

**Pilot: Additional Analyses**

In the pilot study, we included an additional item that asked participants how awkward they felt/would have felt saying “no” to the request. The removal or inclusion of the item does not substantially change the results. Since we did not pre-register or include this item in our pre-registered studies, we report the results of the scale composite without this item in the main text. Here in the SOM, we include the results of the scale composite with the inclusion of this item.

When including the additional item in the scale composite, as predicted and replicating the results from Study 1, participants felt freer to say no in the “how to refuse” (intervention) condition, *M* = 4.55, *SD* = 1.14, 95% confidence interval (CI) = [4.00, 5.10], than in the “right to refuse” (control) condition, *M* = 3.75, *SD* = 1.20, 95% CI = [3.17, 4.33], *t*(36) = 2.12, *p* =.041, *d* = .69, 95% CI = [0.01, 1.36].

Also, the scale item that contained an error in Study 1 also contained an error in the pilot and Study 2. Specifically, the item asked participants, “How pressured did you feel/would you have felt to say “no” to the request?” The item was supposed to ask, “How pressured did you feel/would you have felt to say “yes” to the request?” As before, however, the removal or inclusion of the item does not substantially change the results; thus, in the main text, we report the results without the item included in the scale composite, and here, in the SOM, we report the results with the item included in the scale composite.

When including the scale item in the scale composite, as predicted, participants felt freer to say no in the “how to refuse” (intervention) condition, *M* = 4.92, *SD* = 1.08, 95% confidence interval (CI) = [4.40, 5.44], than in the “right to refuse” (control) condition, *M* = 3.92, *SD* = 1.28, 95% CI = [3.30, 4.54], *t*(36) = 2.60, *p* =.014, *d* = .84, 95% CI = [0.16, 1.53].

Moreover, due to this study's small sample size, we analyzed the behavioral compliance DV using other conservative statistical tests in addition to the chi-squared test reported in the main text. The results of the binary DV remain significant when utilizing more conservative statistical methods. Specifically, the results of a Fisher’s Exact Test, *p* = .019, *OR* = 12.27, 95% CI [1.35, 610.07], and a Chi-Squared Test with Yates’ Continuity Correction, χ^2^ = 5.24, *p* =.022, Φ = 0.37, 95% CI [.05, .69] revealed statistically significant results.

Further, we observe no difference by condition in participants’ self-identified gender, *χ2*(1, *N* = 1) = 2.17, *p* = .141, Φ = 0.24, 95% CI = [-0.08, 0.56], or ethnicity, *χ2*(1, *N* = 3) = 4.10, *p* = .251, Φ = 0.33, 95% CI = [0.01, 0.65]. Additionally, we conducted a likelihood ratio test investigating the effect of gender in addition to the effect of condition on compliance, which revealed that it was not a significantly better predictor, *χ2*(1, *N* = 1) = 0.50, *p* = .478. The results of a separate likelihood ratio test investigating the effect of ethnicity in addition to the effect of condition on compliance also revealed that it was not a significantly better predictor, *χ2*(1, *N* = 3) = 2.05, *p* = .563.

**Study 2: Additional Analyses**

When including the scale item, as predicted and replicating the results from Study 1 and Study 2 pilot, participants felt freer to say no in the “how to refuse” (intervention) condition, *M* = 4.78, *SD* = 1.12, 95% confidence interval (CI) = [4.60, 4.95], than in the “right to refuse” (control) condition, *M* = 4.50, *SD* = 1.34, 95% CI = [4.30, 4.71], *t*(321) = 1.99, *p* =.048, *d* = .22, 95% CI = [.00, .44].

Further, we observe no difference by condition in participants’ knowledge of their right to receive credit for participating in the study despite their refusal or compliance with the request, *χ2*(1, *N* = 323) = 0.91, *p* = .339, Φ = 0.05, 95% CI = [-.06, .16].

Further, we observe no difference by condition in participants’ self-identified gender, *χ2*(1, *N* = 2) = 5.01, *p* = .081, Φ = 0.12, 95% CI = [0.02, 0.23], or ethnicity, *χ2*(1, *N* = 6) = 9.11, *p* = .167, Φ = 0.17, 95% CI = [0.06, 0.28]. Additionally, we conducted a likelihood ratio test investigating the effect of gender in addition to the effect of condition on compliance, which revealed that it was not a significantly better predictor, *χ2*(1, *N* = 2) = 1.91, *p* = .385. The results of a separate likelihood ratio test investigating the effect of ethnicity in addition to the effect of condition on compliance also revealed that it was not a significantly better predictor, *χ2*(1, *N* = 6) = 5.13, *p* = .527.

**Internal Meta-Analysis: Including Pilot Data**

Using the metafor R package (Viechtbauer, 2010), we first meta-analyzed the results of our primary measure of interest: participants’ feelings of freedom to refuse the request. Across all studies, participants in the “how to refuse” (intervention) condition felt freer to say no than participants in the “right to refuse” (control) condition, *d* = .31, *Z =* 3.52, *p* <.001, 95% CI [.14, .48].

Finally, we meta-analyzed the results of the behavioral compliance measure. Crucially, across all studies, participants in the “how to refuse” (intervention) condition complied with the request less often than did participants in the “right to refuse” (control) condition, *OR* = 1.74, *Z =* 2.58, *p* =.010, 95% CI [1.14, 2.64]. Thus, the intervention appears to have a small but significant effect on behavioral compliance, in addition to its robust effect on subjective voluntariness (feelings of freedom to say no).
